# Physical Layer Authenticated Image Encryption for IoT Network Based on Biometric Chaotic Signature for MPFrFT OFDM System

**DOI:** 10.3390/s23187843

**Published:** 2023-09-12

**Authors:** Esam A. A. Hagras, Saad Aldosary, Haitham Khaled, Tarek M. Hassan

**Affiliations:** 1 Faculty of Engineering, Delta University for Science and Technology, Gamasa 35712, Egypt; esam.hagras@deltauniv.edu.eg; 2 Department of Computer Science, Community College, King Saud University, Riyadh 11437, Saudi Arabia; saldosary@ksu.edu.sa; 3 Department of Electronics and Communications, School of Engineering, Edith Cowan University, Perth, WA 6027, Australia; h.khaled@ecu.edu.au

**Keywords:** IoT, physical layer security, dynamic chaotic biometric signature, encryption

## Abstract

In this paper, a new physical layer authenticated encryption (PLAE) scheme based on the multi-parameter fractional Fourier transform–Orthogonal frequency division multiplexing (MP-FrFT-OFDM) is suggested for secure image transmission over the IoT network. In addition, a new robust multi-cascaded chaotic modular fractional sine map (MCC-MF sine map) is designed and analyzed. Also, a new dynamic chaotic biometric signature (DCBS) generator based on combining the biometric signature and the proposed MCC-MF sine map random chaotic sequence output is also designed. The final output of the proposed DCBS generator is used as a dynamic secret key for the MPFrFT OFDM system in which the encryption process is applied in the frequency domain. The proposed DCBS secret key generator generates a very large key space of $2^{2200}$. The proposed DCBS secret keys generator can achieve the confidentiality and authentication properties. Statistical analysis, differential analysis and a key sensitivity test are performed to estimate the security strengths of the proposed DCBS-MP-FrFT-OFDM cryptosystem over the IoT network. The experimental results show that the proposed DCBS-MP-FrFT-OFDM cryptosystem is robust against common signal processing attacks and provides a high security level for image encryption application.

## 1. Introduction

The Internet of Things (IoT) represents a modern internet phenomenon. Device recognition achieves intelligence through establishing or facilitating context-related decisions via the device transceiving information about itself. The rise of cloud computing capabilities leads to an unlimited addressing capacity. The IoT’s purpose is to allow device connectivity with anybody and anything at anytime, anywhere, and via any path/network and service. The IoT can be used in different applications such as transportation, healthcare, power grids, entertainment and smart buildings [1]. Encrypting IoT data before transferring it over wireless networks is one of the simplest and most effective ways to prevent it from being intercepted and altered. Data are converted into an unreadable format by the process of encryption, which can only be decrypted by authorized persons with the right key. IoT services require security to be at the core of everything. Physical layer security (PLS), one of the providing methods for communication security, has attracted a lot of interest from both academics and business since it can provide uncrackable, demonstrable, and quantifiable secrecy. PLS has a significant advantage over encryption since it is not dependent on computational complexity. Consequently, the degree of security attained will not be high, even if the listener has advanced computing capabilities. In contrast to a technique based on encryption, this is founded on the notion that an observer has a constrained computational ability to tackle challenging mathematical puzzles for brief intervals. For the PLS protocol design in IoT, the unique characteristics of IoT, such as cheap cost, wide-range coverage, enormous connection, and varied services impose significant problems [2]. The development of PLS solutions for IoT applications remains difficult despite the success of PLS technique research. The IoT is distinguished by four special characteristics in particular: low cost, broad coverage, high connectivity, and a variety of services. How to design PLS strategies that well match these four features remains an open problem [2].

Recently, [3] have enhanced the dynamics of constellation fluctuations between neighboring frames by utilizing the randomness in the data. The constellation is then dynamically rotated while using analog-based encryption rather than digital-based encryption, which lowers quantization loss and increases robustness to channel phase problems. The others in [4] offer an asymmetric multi-level physical layer security (PLS) scheme in which each transmitted symbol is subjected to two different types of distortion: multi-reception amplitude randomization and channel-based phase distortion. Additionally, the technique streamlines receiver design while providing a significant security advantage for authentic links. The study makes several doable, reasonable, and access-controlled suggestions in [5] for safeguarding the physical layer of the Internet of Things (IoT). The study is still going on with a specific focus on the difficulties with encrypted data. To achieve this goal, a secure approach at the physical layer that provides cryptographic features for usage in conjunction with a flexible RC6 encryption/decryption method is described.

The chaos-based PLS transmission scheme for IoT is introduced in [6]. The suggested approach successfully addresses the concerns with the extremely high PAPR of the OFDM symbols in addition to providing confidentiality of physical layer information transfer by encrypting the Discrete Fourier Transform (DFT) matrix. Additionally, it has no need for additional sideband information and, in theory, has a minimal computing complexity. A physical layer security scheme for OFDM-based IoT systems with compressed sensing is proposed in [7]. The others use a combination of compressed sensing (CS) and OFDM to increase security. Therefore, using compressed sensing, we suggest the PLSSCS physical layer security strategy for OFDM-based IoT systems. By using channel measuring rather than previously collected data, it can alleviate the drawback of key extraction. In [8], the RSA Algorithm and Constellation Encryption Design Based on Chaotic Sequence are introduced. The main goal of this technique is to construct a large number of highly secure encrypted sequences by efficiently combining chaotic sequences and RSA. The precise procedure is to communicate system parameters using the asymmetric RSA algorithm, create a secret sequence using the chaotic sequence’s initial value sensitivity, and then encrypt the original sequence using the secret sequence.

There are many physical layer encryption (PLE) schemes applied to the IoT networks. The key idea of PLE is to exploit the randomness of channels to degrade the received signal quality at the eavesdropper. Three new PLE techniques complement IoT features well and have a lot of promise for use in the future [9]. The noise aggregation and self-encryption [10,11], fountain-coding based secure transmission [12,13,14,15,16,17,18] and Self-Encryption via constellation rotation [19,20,21,22,23,24,25,26,27,28] are different examples of the PLE used in the IoT. OFDM, which has a high spectral efficiency and easy implementation, is used as a self-encryption via the constellation rotation principal. It has been incorporated into different protocols including IEEE 802.11 a/g/n, IEEE 802.16 WiMAX, the frequency domain [29,30,31], data scrambling in the time domain [32], rotation of the modulation symbols [33], and noise-enhanced constellation rotation [34,35] for many of these reasons. Research has looked at using PLE, such as constellation scrambling, to increase the security level of OFDM.

**Figure 1:**
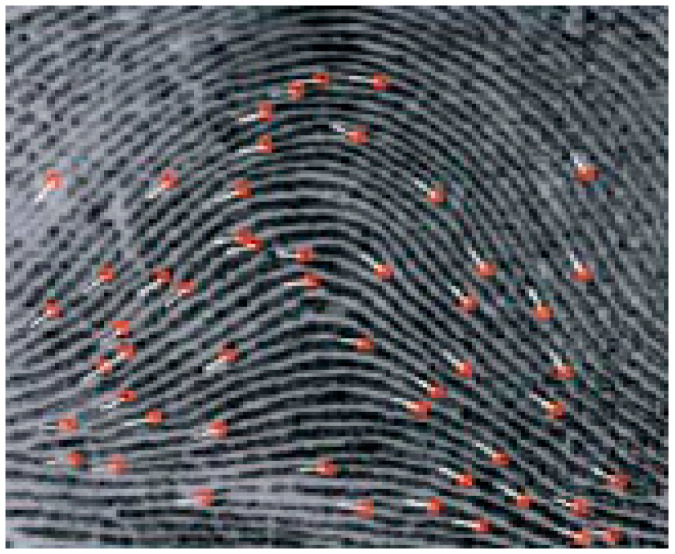
Biometric Fingerprint image.

The majority of IEEE 802.11 Wi-Fi amendments, including 802.11 a, 802.11 g, 802.11 ac, 802.11 n, 802.11 ax, and 802.11 p (the protocol used in vehicle networks) [36,37,38], have embraced OFDM. High-speed Wi-Fi has recently emerged as a viable option for IoT devices due to its compatibility with existing networks. As a result, IEEE 802.11 ah [39] has been proposed as a new Wi-Fi standard for IoT systems. The basic physical layer structure of the transceiver adheres to the conventional design to maintain backward compatibility with access points and clients that support the OFDM physical layer structure despite the fact that this standard offers a number of new and enhanced features to improve power and spectral efficiency [39]. IoT applications can be categorized into two groups: low data rate applications like smart meters and high data rate applications like multimedia IoT. A number of IoT communication protocols, including NB-IoT and 802.11 ah, rely on OFDM as an effective multiple access approach to support the successful operation of high data rate IoT applications [40]. One key feature of IoT systems is their ability to support a variety of legacy and emerging communication protocols, including SigFox, cellular technology, 6LoWPAN (IPv6 Low-power Wireless Personal Area Networks (LoWPAN)), BLE (Bluetooth low energy), ZigBee, RFID (radio frequency identification), NFC (near-field communication), Z-Wave, NB-IoT (Narrow Band IoT), LoRaWAN (long-range wide area network), and Wi-SUN (wireless smart utility network) [41]. There are currently eight major categories of PLS schemes that concentrate on data confidentiality for OFDM systems: channel-based encryption [42], phase encryption [43], permutation [44,45], artificial noise (AN) and artificial fast fading (AFF) [46,47], preamble modulation [48] (Figure 1), power allocation [49], Peak-to-Average Power Reduction (PAPR) encryption [50], the frequency domain [51] and the time domain [52] are two other areas in which these techniques can be used.

Chaos-based physical layer encryption is used in OFDM-based IoT systems to achieve the phase randomization and constellation rotation in the transmitted image in both spatial and transformation domains. An investigation of the Fractional Fourier Transform (FrFT) domains is introduced in [48]. The FrFT parameters are considered as the additional keys for encryption achieving reliable cybersecurity for robust image communication. In [49], multiple fractional order chaotic systems are used in the proposed color image encrypting technique, since using multiple fractional order for image encryption considerably increases the key space and the key sensitivity. A generalization of the FrFT is the multi-parameter fractional Fourier transform (MP-FrFT). Due to the widespread use of MPFrFT in both cryptosystems [50,51,52,53,54,55], more and more academics are becoming interested in it. The authors in [56] introduce the MP-WFRFT and chaotic scrambling-assisted directional modulation technology for improving physical layer security. To realize the power-efficient and security-enhanced wireless transmissions, the directional modulation (DM) technology with multiple parameters weighted-type fractional Fourier transform (MP-WFRFT) and chaotic scrambling (CS) was developed in [56].

In 2023, a new physical layer authentication in wireless networks-based machine learning approaches is introduced in [57]. The purpose of the work given in [57] is to identify and thoroughly compare prior research on physical layer authentication. In addition to demonstrating the most recent PLA techniques, this study examined whether machine learning techniques improved wireless network security performance in physical layer authentication models. Additionally, it pointed out problems and offered lines of inquiry for further study. Researchers and security model creators interested in employing machine learning (ML) and deep learning (DL) methodologies for PLA in wireless communication systems in future research and designs will find this work to be useful. In addition, an application of machine learning techniques in medical data processing based on distributed computing and the IoT is suggested in [58]. Also, in [59], the CNN learning and offloading is used as a hybrid approach for latency and battery lifetime optimization in IoT devices. The main contributions of this research follow:

1. New robust MCC-MF sine map is designed and analyzed.
2. New dynamic chaotic biometric (Digital Fingerprint) signature (DCBS) generator based on the combining the biometric signature and the proposed MCC-MF sine map random chaotic sequence output is also designed.
3. New physical layer authenticated encryption (PLAE) scheme based on the multi-parameter fractional Fourier transform—Orthogonal frequency division multiplexing (MP-FrFT-OFDM) is suggested.

This paper is organized as follows. An introduction is presented in Section 1; Section 2 presents a related preliminary basics. Section 3 presents the proposed MCC-MF sine map, Section 4 presents the proposed DCBS-MP-FrFT-OFDM cryptosystem. Section 4 presents the performance analysis and simulation results discussions of the proposed DCBS-MP-FrFT-OFDM cryptosystem. The following section is the comparison results analysis. Finally, the conclusions and future works are drawn.

## 2. Related Preliminary Basics

### 2.1. Multiple Parameters FrFT

The MPFrFT was presented with its applications and its advantages in signal processing, image encryption and communications in [60]. The $a^{th}$-order continuous FRT of x(t) is given by:

(1) $$\;X_{a}=\int_{-\infty }^{+\infty }x\left(t\right)K_{a}(u,t)dt$$

(2) $$K_{a}\left(u,t\right)=\sqrt{1-jcot\alpha }\cdot e^{j\pi (t^{2}\cot\alpha -tu\csc\left(\alpha \right)+u^{2}\cot\alpha )}$$

where $K_{a}(u,t)$ is the transform kernel and α=aπ/2. The matrix *F* is N×NDFT can be defined as:

(3) $$F_{k,n}=\frac{1}{\sqrt{N}}e^{-j\frac{2\pi }{N}kn},0\leq k,n\leq N-1$$

The DFT matrix F has only four different eigenvalues $\left\{1,-j,-1,j\right\}$. Consider S as a nearly tri-diagonal N×N matrix whose nonzero entries are $S_{n,n}=2\cos2\pi n/N,0\leq n\geq N$ and $S_{n,n+1}$=$S_{n+1,n=1}$ ,0≤n≥N−2, and $S_{n-1,0}$=$S_{0,n-1=1}$. The matrices S and F will have the same eigenvectors if they commute with the matrix F (S·F=F·S) but will not have the same eigenvectors $\lambda_{k}=e\frac{-j\pi k}{2}$. Based on the four different eigenvectors $\left\{1,-j,-1,j\right\}$, the ath-order FrFT matrix of size N×N denoted by $F^{a}$ is defined by [61]:

(4) $$F^{a}=V\Lambda^{a}V^{T}=\left\{\begin{array}{c} \sum_{k=0}^{N-1}\lambda_{k}^{a}v_{k}v_{k}^{T},\;\mathrm{for}N\mathrm{odd}\\ \sum_{k=0}^{N-2}\lambda_{k}^{a}v_{k}v_{k}^{T}+\lambda_{N}^{a}v_{N}v_{N}^{T},\mathrm{for}N\mathrm{even} \end{array}\right.$$

where ${\left(\cdot \right)}^{T}$ denotes the matrix transpose operation, $V=\left[v_{0}v_{1}v_{2}\cdot \cdot \cdot v_{N-2}v_{N-1}\right]$ for N odd and $V=\left[v_{0}v_{1}v_{2}\cdot \cdot \cdot v_{N-2}v_{N}\right]$ for N even, $v_{k}$ is the normalized $k^{th}$-order discrete Hermite–Gaussian-like eigenvector of S, and $\Lambda^{a}$ is a diagonal matrix whose entries are $\lambda_{k}^{a}$ with fractional order a. The MPFrFT can be defined as an extension of the FrFT with multiple parameters by replacing the order a with the vector of fraction orders $\bar{a}$ of length 1×N which are independent fraction orders; then, the MPFrFT denoted by $F^{\bar{a}}$ is defined as:

(5) $$F^{\bar{a}}=V\Lambda^{\bar{a}}V^{T}$$

where $\Lambda^{a}$ is given by:

(6) $$\Lambda^{a}=\left\{\begin{array}{c} diag(\lambda_{0}^{a\left(0\right)},\lambda_{1}^{a\left(1\right)},\cdot \cdot \cdot ,\lambda_{N-1}^{a\left(N-1\right)},\;\;\;\;\;\;\;\;\;\;\;\;\;\;\;for\;N\;odd\\ diag(\lambda_{0}^{a\left(0\right)},\lambda_{1}^{a\left(1\right)},\cdot \cdot \cdot ,\lambda_{N-2}^{a\left(N-2\right)},\lambda_{N}^{a\left(N\right)},\;\;\;for\;N\;even \end{array}\right.$$

In addition, this model of 1D MPFrFT can be modeled as 2D MPFrFT by using two vectors of fraction orders $\bar{a}$ and $\bar{b}$ with lengths of 1×N and 1×M. The two vectors of fraction orders $\bar{a}$ and $\bar{b}$ are independent fraction orders. The 2D MPFrFT can be performed by applying one 1D MPFrFT along rows followed by applying another 1D MPFrFT along columns. The 2D MPFrFT denoted by $F^{\left(\bar{a,}\bar{b}\right)}$ is defined as [61]:

(7) $$F^{\left(\bar{a,}\bar{b}\right)}=V\Lambda^{\left(\bar{a,}\bar{b}\right)}V^{T}$$

Then, the 2D MPFrFT of a 2D input P of size M×N can be defined in a row–column scheme as:

(8) $$F^{\left(\bar{a,}\bar{b}\right)}\left[P\right]=F^{\left(\bar{a}\right)}PF^{\left(\bar{b}\right)}$$

The properties of the MPFrFT are given in [62]. The main advantage of the 2D MPFrFT is that the two vectors of fraction orders $\bar{a}$ and $\bar{b}$ with lengths of 1×N and 1×M can be used as an additional secret key for secure applications.

### 2.2. Biometric Authenticated Secret Key

A fingerprint can be used as a biometric property to extract digital data using a variety of methods, such as a block-based approach to create a feature vector [62]. With the help of this feature vector, code words can be created that are sufficiently random and large to be employed. The procedure includes the following steps: feature extraction, straight line attribute calculation, straight line attribute obfuscation, and production of a biometric binary string. Then, from the fingerprint image, we extract the minute points, core points, and delta points. If P is a collection of minute points, then $p\left(x,y\right)$ stands for a minute point’s coordinate. A collection of minor points is denoted by the notation point $p=\left\{p_{1}\left(x_{1},y_{1}\right),p_{2}\left(x_{2},y_{2}\right),\ldots ,p_{k}\left(x_{k},y_{k}\right)\right\}.$ Miniscule points are represented by $p_{i}(x_{i},y_{i}),$ i=1,2,…k. The core point is then represented as $Cp(x_{c},y_{c})$, where $x_{c}$ is the x-coordinate and $y_{c}$ is the y-coordinate of the discovered core point “Cp“from the input fingerprint picture. Finally, when a delta point is found in a fingerprint picture, it is represented as $D_{p}(x_{d},y_{d})$, where $x_{d},$ is the discovered delta point’s x-coordinate and $y_{d}$ is its y-coordinate. Divide the image into small blocks and compute the straight-line properties between the points in the set ‘P’. The fingerprint image ‘I’ will be divided into a number of tiny blocks, each measuring m×m pixels, with I=p×q of all blocks.

Using all the blocks, we determine the straight-line properties when computing all straight lines from a block’s minutiae point ($p_{k}$), which stands for the block in the $i^{th}$ row and $j^{th}$ column of $I_{ij}$ as a reference block for all other blocks’ minutiae points. Compute the length and angle of each straight line, using the Euclidean distance for length (*l*i) and the *x*-axis for angle $\left(a_{i}\right)$. Let $F_{B}$ represent a collection of straight-line lengths and angles for all blocks, $F_{B}=\{(l_{1},a_{1}),(l_{2},a_{2}),\ldots ,(lzb,azb)\}$. Find the block $I_{lm}$ that contains the core point ($C_{P}$), compute all straight lines that connect the core point ($C_{P}$) to all other minutiae points of neighboring blocks, and then extract the core and delta points from image I. Let $F_{C}$ denote a set of lengths and angles of straight lines, where the size of the $F_{B}$ is $z^{b}$. Finally, the extracted minutiae attributes contain three fields per minutiae: the x-coordinate ([1, 511]), y- coordinate ([1, 511]) and orientation θ ([0, 359]); the three parameters (x.y.θ) are used as a biometric minutia [63,64,65]. In [64], a high-performance fingerprint scanner and a recognition engine are both included in the FS83 serial Fingerprint Authentication Module (FS83-sFAM), which is used in order to generate 2072 bytes from three samples of different fingerprints of one user. The resultant bits are represented in hexadecimal format, which is used in authenticated and secret key generation. The biometric fingerprint image is shown in Figure 1.

## 3. Proposed Multi-Cascaded Chaotic Modular Fractional Sine Map (MCC-MF Sine Map)

The cascade chaotic system (CCS) is a general 1D chaotic framework for creating new nonlinear chaotic systems using any two 1D chaotic maps as seed maps; it was first introduced in [66]. Zhongyun et al. also suggested a dynamic parameter-control chaotic system (DPCCS) [67] based on the concept of the CCS. The DPCCS has a simple architecture that uses the control map’s output to dynamically modify the seed map’s parameters. CCS and DPCCS have straightforward hardware implementation, simple structures, and wildly unpredictable behavior. In this section, a new MCC-MF sine map is introduced and analyzed. The development of discrete fractional calculus allowed for the effective incorporation and capture of memory effects in nonlinear discrete temporal systems. Complex features are seen in chaotic systems with a fractional order. Assume that a sequence ρ(n) is given and the isolated time scale ${ℵ}_{a}$ is represented in terms of the real valued constant τ as {τ,τ+1,τ+2,…,} such that $\rho :{ℵ}_{\tau }\to R.$ The difference operator is denoted by Δ, where $\Delta \rho \left(n\right)=\rho \left(n+1\right)-\rho \left(n\right)$. Then, we summarize some of the basic definitions related to discrete fractional calculus as follows:

The fractional sum of order α (α>0) is given by [68]:

(9) $$\Delta_{\tau }^{-\alpha }\rho \left(t\right)=\frac{1}{\Gamma \left(\alpha \right)}\sum_{m=\tau }^{t-\alpha }\frac{\Gamma \left(t-m\right)}{\Gamma \left(t-m-\alpha +1\right)}\rho \left(m\right),t\in {ℵ}_{\tau +\alpha }.$$

The Caputo-like delta difference of order α is defined by [68]:

(10) Δτα Cρt=Δτ−n−αΔnρt=1Γn−α∑m=τt−n−αΓt−mΓt−m−n+α+1Δnρm,t∈ℵτ+n−α,n=α+1.

The delta fractional difference equation of order α is represented by [69]:

(11) Δτα Cρt=f(t+α−1,ρ(t+α−1)),

The equivalent discrete fractional integral is given by [70]:

(12) $$y\left(l\right)=\rho_{0}\left(t\right)+\frac{1}{\Gamma (\alpha )}\sum_{m=\tau +n-\alpha }^{t-\alpha }\frac{\Gamma \left(t-m\right)}{\Gamma \left(t-m-\alpha +1\right)}\times f(m+\alpha -1,\rho (m+\alpha -1)),t\in {ℵ}_{\tau +n}.$$

Note that the initial iteration in this case is [71]:

(13) $$\rho_{0}\left(t\right)=\sum_{k=0}^{n-1}\frac{\Gamma \left(t-\tau +1\right)}{k!\Gamma \left(t-\tau -k+1\right)}\Delta^{k}\rho \left(\tau \right).$$

The non-modular fractional sine chaotic map is given by [72]:

(14) $$x\left(i\right)=x\left(0\right)+\frac{1}{\Gamma v}\sum_{j=1}^{n}\frac{\Gamma (i-j+v)}{\Gamma (i-j+1)}r_{1}\sin\left(\pi r_{1}\right)$$

**Figure 2:**
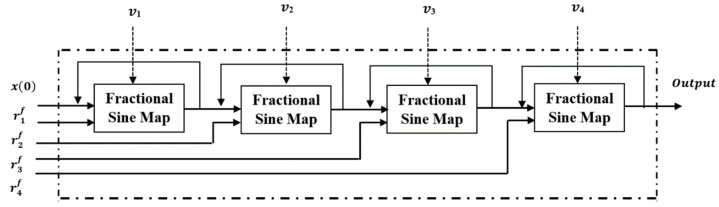
Proposed MCC-MF sine map block diagram.

The proposed MCC-MF sine map is designed based on the concept of a cascade chaotic system. The fractional chaotic map is given by [68,69,70,71,72] and the final mathematical model is given by:

(15) $$x\left(i\right)=x\left(0\right)+\frac{1}{\Gamma \;v_{i}}\sum_{j=1}^{n}\frac{\Gamma (i-j+v_{i})}{\Gamma (i-j+1)}r_{1}\sin\left(\pi r_{2}\sin\left(\pi r_{3}\sin\left(\pi r_{4}\sin\left(\pi x(j-1)\right)\right)\right)\right)$$

where $r_{1},r_{2},r_{3}$ and $r_{4}$ are the control parameters and $x\left(0\right)$ is the initial condition of the proposed map. Using more than one parameter of the sine map gives a high Lyapunov exponent (LE) value, high chaotic range and a large key space. The block diagram of the proposed MCC-MF sine map is shown in Figure 2. The proposed MCC-MF sine map consists of four fractional chaotic sine maps connected in concatenated form with different secret parameters. The modular function is used to improve the chaotic property based on the continuity of the map output.

**Figure 3:**
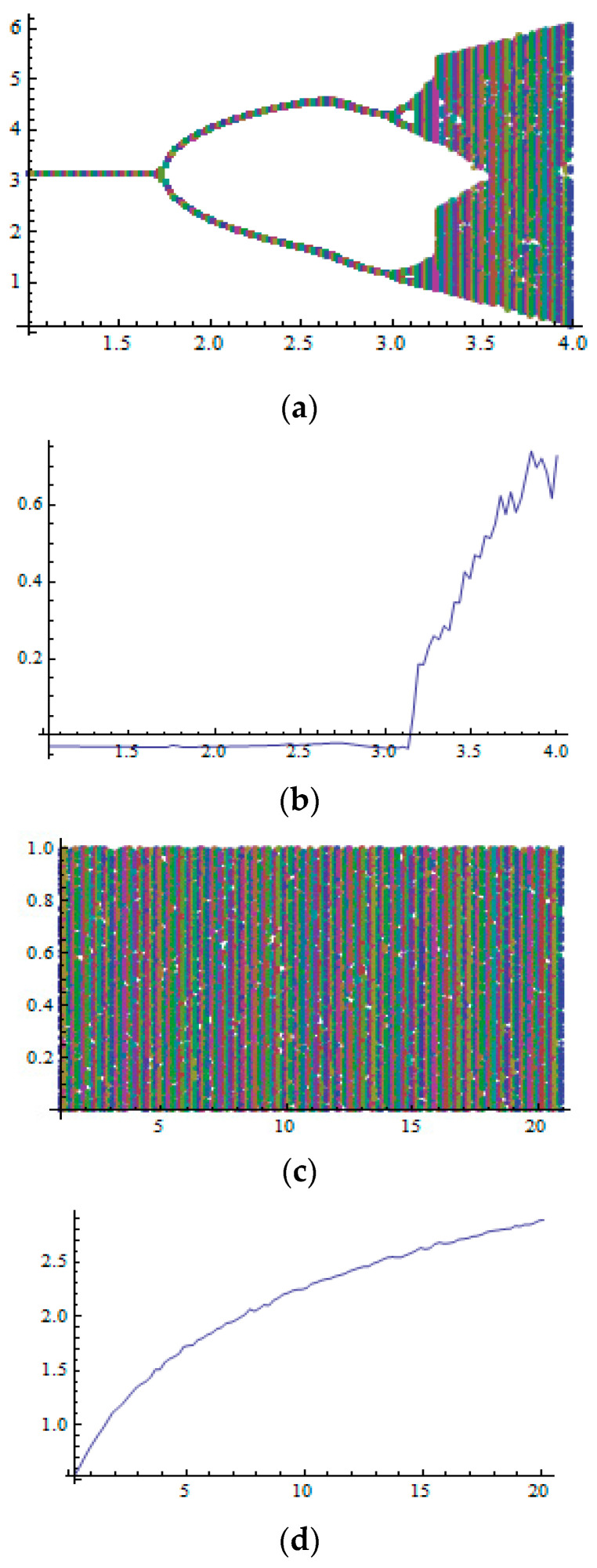
(**a**) BD of the conventional non-modular FSCM. (**b**) LE of the conventional non-modular FSCM. (**c**) BD of the proposed MCC-MF sine map. (**d**) LE of the proposed MCC-MF sine map.

The effect of the fractional order on the chaotic map can be shown in Figure 3, where Figure 3a–d describe the output series, the bifurcation diagram (BD), and the Lyapunov value.

The NIST test suit, which consists of 16 statistical tests, is used to determine the randomness of the proposed MCC-MF sine map. These tests determine whether or not the created sequence is random. These tests’ primary reliance is on the probability value (*p* value). The significance level, which is the line separating the rejection and non-rejection regions, compares the *p*-value. The significant level in NIST is set at 0.01. If the *p*-value is less than or equal to 0.01, the sequence is not random and is rejected; if it is greater than 0.01, the sequence is random and accepted. The proposed MCC-MF sine map’s binary sequence of $10^{6}$ bits is examined using SP800-22 [73], and the results are shown in Table 1.

## 4. Proposed Secure MP-FrFT-OFDM Cryptosystem

Due to their effective use of network resources and bandwidth, ability to accommodate a range of mobility scenarios, and ability to deliver high data rates, OFDM systems have demonstrated widespread success in many wireless communication applications. Thus, it is anticipated that OFDM will continue to be a crucial enabling technology in present and future systems, including 5Gs [42]. In order to deal with inter-channel interference (ICI) and inter-symbol interference (ISI) problems and permit simultaneous data transmission via band-limited channels, OFDM was first presented in the middle of the 1960s [48]. Wide frequency selective channels are, in theory, divided into a number of small, flat fading sub-bands by OFDM. Despite the fact that OFDM sub-bands are made orthogonal and independent of one another, a guard band known as the cyclic prefix (CP) is necessary to lessen the impacts of ISI and ICI. Instead of employing an empty guard space, the idea of a CP is based on adding a cyclic extension to the symbol itself. In the suggested encryption scheme, authenticated biometric features are utilized as the biometric secret key generation with the proposed MCC-MF sine map chaotic secret key generation in order to design a DCBS generator for the MPFrFT OFDM image encryption.

### 4.1. Proposed DCBS Generator

**Figure 4:**
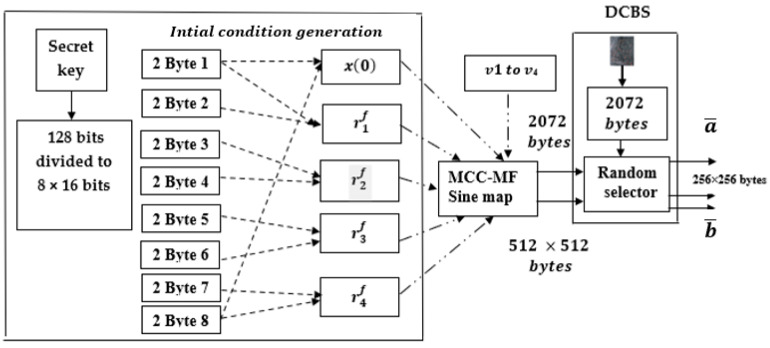
Proposed DCBS generator block diagram based on MCC-MF sine map.

The design of the proposed DCBS generator is based on the secure fractional number sequence generated from the proposed MCC-MF sine map and the biometric fingerprint minutiae generated from the FS83 s-FA Module [62]. We assumed that the FS83 s-FA Module generated a sequence “T” ∈ [1, 256] with a length of 2072 bytes. The block diagram of the proposed DCBS generator is shown in Figure 4. As shown in Figure 4, the proposed DCBS generator consists of 128 secret keys, an initial condition generation, and the proposed MCC-MF-sine map. The secret key is used for initial condition generation for the proposed MCC-MF-sine map with the fractional secure parameters ($v_{1}\;to\;v_{4}$) in order to generate 2072 bytes and 512 × 512 bytes. The output of the MCC-MF-sine map is used as an input for the DCBS generator to produce two vectors $\bar{a}$ and $\bar{b}$ of sizes (1 × 256) and 256 × 256 bytes for the encryption and authentication process.

1. The secret key (SK) of 128 bits represented by 32 hexadecimal digits “C2250EA6637F5AFAAF0654 9CCD16220A” is used to combined the biometric signature with the fractional number sequence generated from the proposed MCC-MF sine map.
2. The secret key is divided into eight sections to generate the initial conditions and the different control parameters of the proposed MCC-MF sine map. All secret parameters and the initial condition are $10^{-15}$ decimal precision.

3. The first eight hexadecimal digits ($k_{s}^{1}$ ) and the last eight hexadecimal digits number eight $\left(k_{s}^{8}\right)$ are used to generate the fractional initial condition of the proposed MCC-MF sine map as:
  (16) $$x_{0}=(\frac{hex2dec\left(k_{s}^{1}\right)}{hex2dec\left(k_{s}^{8}\right)}\mathrm{mod}1)$$

4. The first ($k_{s}^{1}$ ) and the second ($k_{s}^{2}$) eight hexadecimal digits are used to generate the first fractional secret control parameter ($r_{1}^{f}$) as:
  (17) $$r_{1}^{f}=(hex2dec\left(k_{s}^{1}\right)+hex2dec\left(k_{s}^{2}\right)\;*\;10^{-6})\;\mathrm{mod}\;20$$

5. The next three fractional secret control parameters are given as:

(18) $$r_{2}^{f}=(hex2dec\left(k_{s}^{3}\right)+hex2dec\left(k_{s}^{4}\right)\;*\;10^{-6})\;\mathrm{mod}\;20$$

(19) $$r_{3}^{f}=(hex2dec\left(k_{s}^{5}\right)+hex2dec\left(k_{s}^{6}\right)\;*\;10^{-6})\;\mathrm{mod}\;20$$

(20) $$r_{4}^{f}=(hex2dec\left(k_{s}^{7}\right)+hex2dec\left(k_{s}^{2}\right)\;*\;10^{-8})\;\mathrm{mod}\;20$$

6. The proposed MCC-MF sine map given in Equation (15) is iterated *t* = 512 × 512 × 8 times by using the generated ${x_{0},r_{1}^{f},r}_{2}^{f},r_{3}^{f},r_{4}^{f}$ secret parameters and the fractional secure parameters ( $v_{1}\;to\;v_{4}$).

7. Ignore the first 1000 bits to prove the chaos property of the generated chaotic sequence. In addition, select the last 2072 bytes of the generated chaotic sequence.
8. Concatenate the chaotic sequence output (2072 bytes) with the 2072 bytes of the biometric signature to generate the dynamic chaotic biometric signature (DCBS).
9. Finally, randomly select 256 × 256 bytes from the iterated chaotic sequence 512 × 512 bytes for the diffusion process by Xoring with the original image and select the two different 256 vectors ($\bar{a}\;and\;\bar{b}$ ), which are used as the secret multi-parameters for the confusion process in the MPFrFT OFDM transform.

### 4.2. Secure MP-FrFT-OFDM Based on MCC-MF Sine Map and DCBS Generator

The concept of OFDM is used in the physical layer communication based on Fast Fourier Transform (FFT). Also, the Fractional Fourier Transform (FrFT) is used in the OFDM system. The FrFT used only one parameter for the phase shift in the FFT which converts the FFT to FrFT. In the multi-parameters, FrFT used a vector of secret fractional values (0 to 1) with a length equal to the length of the FrFT for the OFDM system. In addition, the encryption process is applied in the frequency domain based on the OFDM system, which is the standard modulation used in the physical layer (IEEE 802.11 a/g/n). In this section, we suggested using the MPFrFT and the FrFT instead of the standard FFT in OFDM. The FFT–OFDM is only a multi-carrier transmission system. By using the FrFT instead FFT in OFDM, only one fraction order is used to change the phase in the output of the FrFT, which cannot satisfy any encryption properties. In this paper, the MPFrFT is used with the OFDM, where the MPFrFT has multi-parameters (ordered) equal to the length of the FrFT used, the length of MPFrFT can be 256, which means that the 256 parameters can be used as a secret key (secure multi-phase changing parameters). So, the encryption can be applied in the frequency domain without any additional equipment.

**Figure 5:**
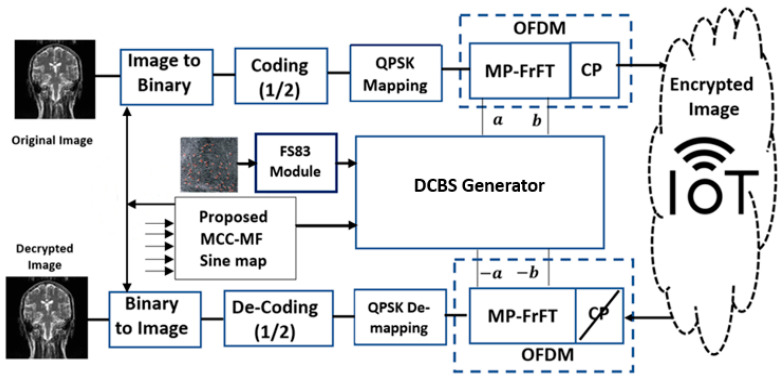
The proposed secure MP-FrFT-OFDM based on MCC-MF sine map and DCBS generator.

The framework of the proposed secure MP-FrFT-OFDM based on MCC-MF sine map and DCBS generator is given in Figure 5. The first step in the proposed MPFrFT OFDM image encryption based on the MCC-MF sine map and DCBS generator key is changing the input image into a binary format of (d=256×256×8) bits, d∈{0,1}. The second step involves applying the convolutional coding with a code rate (R=1/2) to the image data bits as an error-correcting code. The coded data sequence is mapped onto the QPSK-modulated symbols. Based on the proposed DCBS generator, the secret multi-parameters are generated for the MPFrFT OFDM image encryption process; finally, the cyclic prefixes are added to the output of the MPFrFT OFDM encrypted data. In the receiver, the inverse processes are used. The three steps of the proposed cryptosystem will be discussed. The proposed secure MP-FrFT-OFDM based on MCC-MF sine map and DCBS generator is shown in Figure 5.

### 4.3. Authenticated Encryption Scheme

The ciphering technique’s steps can be summed up as follows:

1. Read the image that was entered.
2. Convert the input image into binary format.
3. The first encryption step started with the diffusion process by Xoring, which converts the binary image data of 256 × 256 × 8 bits with the select random iterated chaotic sequence of 256 × 256 × 8 bits.
4. Apply convolutional coding for the diffusion 256 × 256 × 8 bits.
5. Apply QPSK mapping.
6. The second encryption step is the confusion process, applying the inverse MPFrFT based OFDM modulation based on the two secret fractional parameter vectors *a* $=256\;\mathrm{bytes}(a_{1},a_{2},\cdots ,a_{256})$

and b=256 bytes $\left(b_{1},b_{2},\cdots ,b_{256}\right)$ as follows:

(21) $$a=0.9+\frac{1}{100}\sum_{i=1}^{i=256}a_{i}$$

(22) $$b=0.9+\frac{1}{100}\sum_{i=1}^{i=256}b_{i}$$

7. Add the cyclic prefix (CP) to the output of the secure MP-FrFT.
8. Send the encrypted image across an IoT channel to the recipient side.

### 4.4. Authenticated Decryption

The deciphering technique’s steps can be summed up as follows:

1. Receive the authenticated encrypted image data.
2. Remove the cyclic prefix (CP) from the received secure MP-FrFT.
3. The first step in the decryption is the de-confusion by applying the inverse MP-FrFT-based OFDM on the encrypted image based on the inverse two secret fractional parameter vectors −a and −b as follows:

(23) $$-a=-(0.9+\frac{1}{100}\sum_{i=1}^{i=256}a_{i})$$

(24) $$-b=-(0.9+\frac{1}{100}\sum_{i=1}^{i=256}b_{i})$$

4. Apply QPSK de-mapping.
5. Apply convolutional de-coding for the diffusion 256 × 256 × 8 bits.
6. Convert the authenticated encrypted image into binary format.
7. The second step in the decryption step is the de-diffusion by Xoring of the authenticated encrypted binary image data of 256 × 256 × 8 bits with the select random iterated chaotic sequence of 256 × 256 × 8 bits.
8. Apply the required analysis.

Examining the impact of noise, information entropy, visual inspection, histograms, assaults, differential, and encryption quality metrics, the effectiveness and security of the proposed system are examined. The recommended picture encryption approach maintains a good security quality, according to all numerical results.

## 5. Performance Analysis and Results

Key space analysis, UACI, NPCR, neighboring pixel correlation analysis, and histogram analysis are only a few of the statistical and security analysis techniques used. A CT scan of the brain (medical) and other images are chosen with different standard gray-scale test images such as Cameraman, Peppers and Lena for system simulation. The test image is 256×256 pixels. The OFDM parameters include the following: the total number of OFDM sub-carriers is denoted by ${(N}_{sc}=256)$, the FFT length is set to a 256 bit length, and the CP is set to a 32 bit length. The suggested system performance is tested under the AWGN channel effect at zero mean μ=0 and at different values of noise variances, $\left(\sigma^{2}=0.01,0.05,0.10,0.15,0.20\right)$. Also, the proposed system is tested under different signal processing attacks as Salt and Pepper noise and Speckle noise. Analysis of the histogram, neighboring pixel correlation, key space, NPCR, and UACI tests are examples of statistical and security analysis. The multi-secure simulation parameters are displayed in Table 2. The suggested authenticated secure image transmission system simulation parameters are also shown in Table 2 and Table 3.

### 5.1. Visual Quality Metrics

Remarkable indicators used in studying the encryption robustness are represented as the Key Performance Indicators (KPIs) for the proposed system. Visual quality inspection is measured in terms of *BER* performance and PSNR as a clarity investigation performance as well as statistical measures to evaluate the degree of encryption quantitatively. The visual quality metrics for the proposed scheme are measured in terms of BER performance and *PSNR* performance in the form $E_{b}N_{0}vs.BER,$ and $E_{b}N_{0}vs.PSNR$ as a visual testing for the received image. Different $E_{b}N_{0}$ values between 0 and 18 dB are used to calculate the *PSNR* values of the received image. Bit Error Rate (BER) is a signal quality metric that evaluates the performance of the entire system, including the transmitter, the receiver, and the medium used to connect them. *BER* is defined as the ratio of the number of bits received in error due to interference, noise, or other problems to the total number of bits received. In [24], the *BER* simple formula is defined.

(25) $$BER=\frac{\mathrm{Number}\mathrm{of}\mathrm{bit}\mathrm{errors}}{\mathrm{Total}\mathrm{number}\mathrm{of}\mathrm{transferred}\mathrm{bits}}$$

(26) $$PSNR=10\times {log}_{10}\frac{f_{max}^{2}\times M\times N}{\sum_{i=1}^{M}\sum_{j=1}^{N}{\left(I\left(i,j\right)-I^{\prime }\left(i,j\right)\right)}^{2}}$$

The *PSNR* ratio, which is measured in decibels (dB), is regarded as a visual quality metric test of the reconstructed (decrypted) image compared to the original transmitted image [25]. The quality of the produced image will be better the higher the *PSNR* value. Here, $f_{max}^{2}$ denotes the highest pixel value possible, I(i,j) denotes the original image pixel, $I^{’}(i,j)$ denotes the received image pixel values, (*M* × *N*) denotes the image size, and all other variables are equal. Various images with a resolution of 256×256 pixels are used to test the simulation analysis for the proposed authenticated secure image transfer technique. The AWGN channel is a well-known model to indicate various random processes seen in nature; it contains a uniform power across the whole frequency band. Starting with a CT brain medical image, the proposed system behavior is examined under the AWGN channel effect at zero mean μ=0 and over certain ranges of noise variances, $\left(\sigma^{2}=0.01,0.05,0.10,0.15,0.20\right),$ as shown in Table 4.

**Figure 6:**
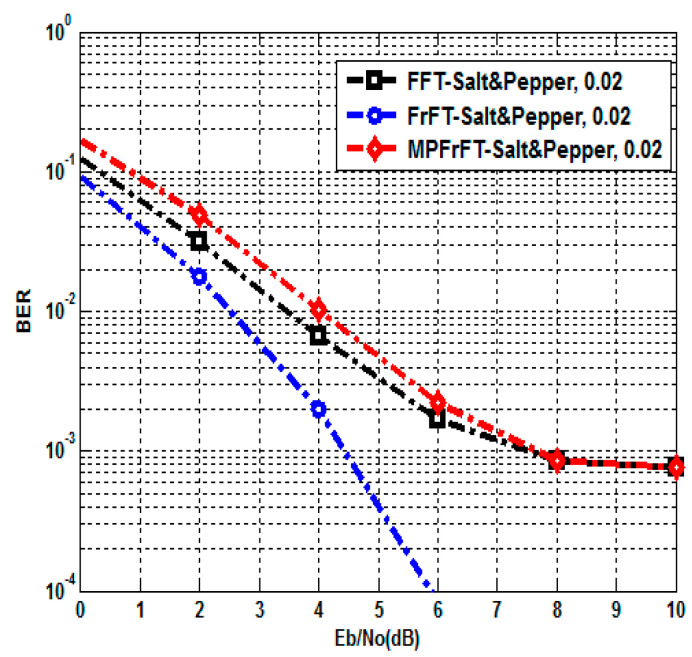
BER of FFT, FrFT, MPFrFT OFDM over Salt and Pepper noise attack, d=0.02.

**Figure 7:**
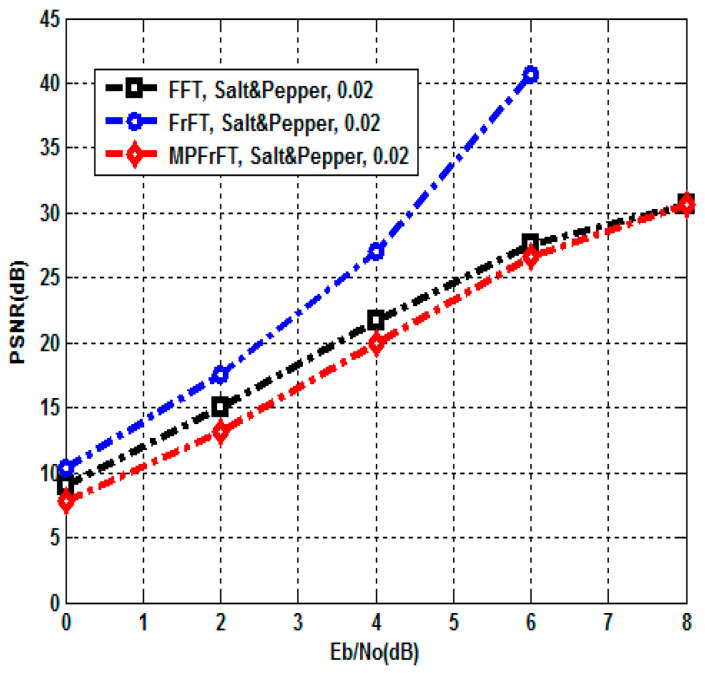
PSNR of FFT, FrFT, MPFrFT OFDM over Salt and Pepper noise attack, d=0.02.

A Salt and Pepper noise attack is when a certain amount of the pixels in the image are affected by an impulse type of noise represented by either black or white dots (hence the name of the noise), which can significantly deteriorate the quality of an image [3]. It can be used to model defects in the transmission of the image. The proposed authenticated secure image transmission scheme system is examined under Salt and Pepper noise attack; here, the noise density d=0.02. The BER and PSNR performances of the proposed FFT,FrFT and MPFrFT coded OFDM schemes are tabulated at different values of $E_{b}N_{0}(0to10dB)$ in Table 5 and Table 6. The results given in Table 5 are plotted in Figure 6 and Figure 7, respectively. In Figure 6, at $E_{b}N_{0}=8dB,$ the FFT-OFDM BER performance is $8.60\times 10^{-4}$, the FrFT-OFDM BER performance is $8.21\times 10^{-4}$ and the MPFrFT-OFDM BER performance is $8.59\times 10^{-4}$. In Figure 6, at $E_{b}N_{0}=8dB,$ the FFT-OFDM PSNR performance is 30.65dB, the FrFT-OFDM PSNR performance is 30.8548dB and the MP-FrFT-OFDM PSNR performance is 30.6579dB. The proposed FrFT-OFDM system gains PSNR performance improvement by about 0.1969dB compared with the proposed MPFrFT-OFDM system, but a large key space is achieved by the MPFrFT OFDM system than the FrFT-OFDM system.

Different $E_{b}N_{0}$ values 2,8 and 16dB are chosen in Table 7 in order to highlight the visual quality metric performance of the proposed systems under the Salt and Pepper noise effect. In medical ultrasound imaging, Speckle is a granular interference that inherently exists in and degrades the quality of the medical images. It results from the coherence of backscattered signals from various distributed targets [4].

**Figure 8:**
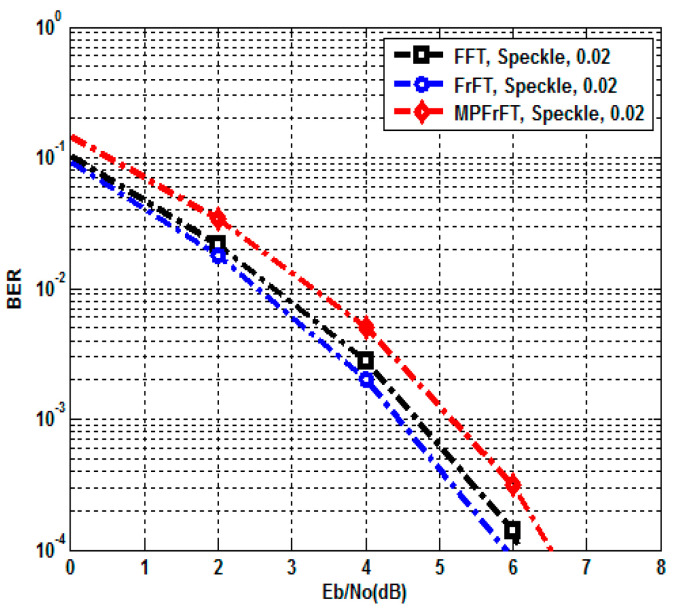
BER performance, FFT, FrFT, and MPFrFT OFDM over Speckle noise attack, $\delta^{2}=0.02$.

**Figure 9:**
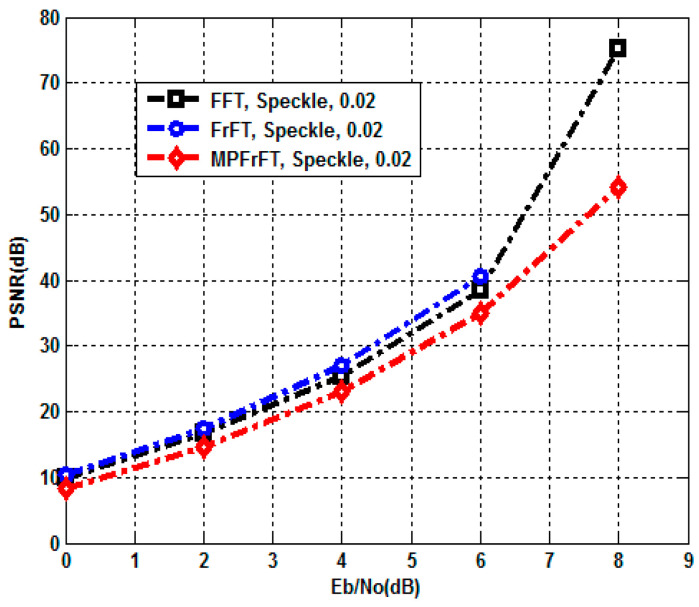
PSNR performance, FFT, FrFT, and MPFrFT OFDM over Speckle noise attack, $\delta^{2}=0.02$.

Table 8 presents both BER and PSNR metrics performance for the proposed FFT,FrFT and MPFrFT coded OFDM systems at different values of $E_{b}N_{0}(0dBto16dB)$ under Speckle noise attack. Speckle noise is represented as a multiplicative noise to the brain medical test image, using uniformly distributed random noise with zero mean, μ=0 and variance, $\delta^{2}=0.02$. The results in Table 8 are plotted in Figure 8 and Figure 9 in order to clarify the Speckle noise attack effect on the proposed authenticated secure medical image transmission schemes. BER calculations were obtained for the introduced FFT,FrFT and MP−FrFT coded OFDM systems at $E_{b}N_{0}=8dB$; the BER values are $3.70\times 10^{-8}$, 0, and $3.11\times 10^{-4}$ respectively. At the same $E_{b}N_{0}=8dB$, the PSNR performance values are 75.1229dB, InfdB, and 54.1514dB respectively. Then, at $E_{b}N_{0}\geq 8.50dB$, the proposed systems provide the highest BER and PSNR performance (0BERandInfPSNR). Speckle noise is represented as a multiplicative noise to the brain medical test image, using uniformly distributed random noise with zero mean, μ=0 and variance, $\delta^{2}=0.02$. In addition, Table 9 shows the BER and PSNR performance for FFT, FrFT, and MPFrFT coded OFDM over Speckle noise attack at $\delta^{2}=0.02$ at $E_{b}N_{0}=2,8,8.50dB$.

### 5.2. Encryption Quality Metrics

Encryption quality metrics for the proposed scheme are measured using deferential attack analysis, correlation analysis, histogram analysis, entropy analysis and key space analysis.

#### 5.2.1. Deferential Attack Analysis

The number of pixels change rate (also known as the NPCR) and unified average changing intensity (also known as the UACI) are two frequently used tests that were used to evaluate the sensitivity of the encrypted image. To strengthen resistance to the differential attack, each small change to the plain image should result in a significant disruption of the cipher image. Consider $C_{1}$ and $C_{2}$, two cypher pictures for two planar images $p_{1}$ and $p_{2}$, which only differ by one pixel. $C_{1}(i,j)$ and $C_{2}(i,j)$ are the gray-scale pixel values of the two images $C_{1}$ and $C_{2}$, respectively. The *NPCR* and *UACI* are defined as [13]:

(27) $$NPCR=\frac{\sum_{i=1}^{N}\sum_{j=1}^{M}D(i,j)}{M*N}*100\%$$

(28) $$UACI=\frac{1}{M*N}\frac{\sum_{i=1}^{N}\sum_{j=1}^{M}\left|C_{1}\left(i,j\right)-\left.C_{2}(i,j)\right|\right.}{2^{n}-1}*100$$

where D(i,j) is an identical-sized bipolar array to the cypher picture that is described as:

(29) $$D\;(i,\;j)=\left\{\begin{array}{c} 1\;if\;C_{1}{\left(i,j\right)\neq C}_{2}(i,j)\\ 0\;if\;C_{1}{\left(i,j\right)=C}_{2}(i,j)\; \end{array}\right.$$

In the simple images, $p_{i}$ is the value of the initial pixel. Without modifying any other values, it is changed to $p_{i}$ = ($p_{i}$ + 100) mod256 to obtain a second image and encrypt the two images in order to calculate the NPCR and UACI values of the two encoded images. Table 10 and Table 11 display the findings of the NPCR and UACI for the proposed MP-FrFT, FrFT-coded OFDM using Cameraman, Peppers and Boat standard gray-scale test images. Table 10 and Table 11 shows the NPCR and UACI the comparison among the proposed MPFrFT and FrFT-coded OFDM using Cameraman, Peppers and Boat standard gray-scale test images 

#### 5.2.2. Correlation Analysis

Correlation is defined as a statistical relationship that measures the relativity between two variables. The correlation between the original image and encrypted image is measured between two vertically adjacent pixels: a plain image/cipher image, respectively [4]. If the correlation coefficient values are closer to 1, it reflects highly dependent variables between the original and deciphered image (i.e., good decryption quality). If the correlation coefficients are closer to 0, it refers to highly independent variables between the original and cipher image (i.e., totally different, no features between original image and encrypted one, high-quality encryption algorithm). Smaller values of the correlation coefficients assess a successful encryption/decryption process. The correlation between original and encrypted images for the proposed systems MPFrFT and FrFT are tabulated in Table 12. These correlation coefficient values ensure the immunity of the proposed schemes. The correlation coefficient $\left(r_{xy}\right)$ is defined as [3]:

(30) $$r_{xy}=\frac{Cov(x,y)}{\sigma_{x}\times \sigma_{y}}$$

(31) $$Corr=\frac{\sum_{i=1}^{N}\left(x_{i}-E\left(x\right))(x_{y}-E\left(y\right)\right)}{\sqrt{{\left(x_{i}-E\left(x\right)\right)}^{2}}\times {\sqrt{{\left(x_{i}-E\left(x\right)\right)}^{2}}}^{,}}$$

where $E\left(x\right)=\frac{1}{N}\times \sum_{i=1}^{N}x_{i}$, x,y are the gray-scale pixel values of the source and enciphered images.

#### 5.2.3. Histogram Analysis

The definition of a histogram is a statistical graphical distribution of each discrete intensity level (also known as a “gray level”) in a digital image into user-specified ranges. It displays the gray scale, the density of the gray-level distribution, the average luminance of an image, picture contrast, and so on. The histogram’s horizontal axis displays the potential intensity values, while the vertical axis displays the number of pixels for each of these intensities [5]. The proposed MPFrFT and FrFT-coded OFDM ciphering approaches reflect identical histograms of the relevant source images, according to the reported histogram analysis. As a result, an encrypted image’s statistical metrics are the same as those of the matching source image. Table 13 shows the histogram analysis for the proposed MPFrFT-coded OFDM using Cameraman, Peppers and Boat standard gray-scale test images.

#### 5.2.4. Key Space Analysis

**Figure 10:**
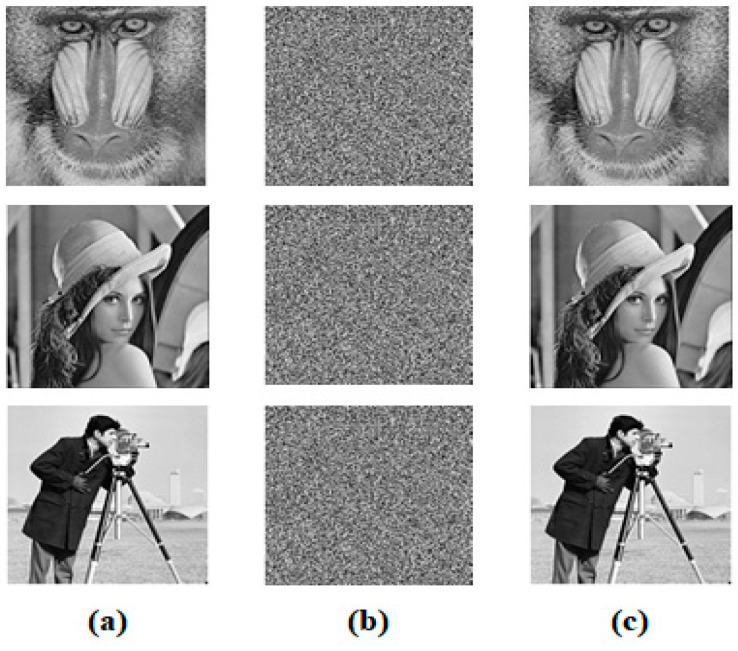
Encryption and decryption results of the gray images Baboon, Lena, and Cameraman. (**a**) the original images, (**b**) the encrypted images, (**c**) the decrypted images.

The total number of unique keys that can be utilized in the encryption process is calculated by the key space. If the length of each initial value or control parameter is set to 16 decimals, the secret keys for the proposed encryption consist of eight initial values ($x_{0}^{1}$) valid in the range of [0, 1] and four control parameters $(r^{1}$,$r^{2}$,…,$r^{4})$ valid in the range of 0.1 to 20. The key space determines the total number of distinct keys that can be used in the encryption process. The secret keys for the suggested encryption consist of eight starting values ($x_{0}^{1}$) valid in the range of [0, 1] and four control parameters $(r^{1}$,$r^{2}$,…,$r^{4})$ valid in the range of 0.1 to 20 if the length of each initial value or control parameter is set to 16 decimals. It is possible to determine the entire complexity (total key space) as follows: $10^{15}\times 10^{15}\times 10^{15}\times 10^{15}\times 10^{15}$ = $10^{4\times 15}$=$10^{60}$. The key space of an image of size 256 × 256 is 256 × 256 × $2^{8}$ = 4×$10^{5}$. In addition, the multi-parameter $\bar{a}$ and $\bar{b}$ has a key space for $\bar{a}$ = $10^{15\times 256}\;$ and for $\bar{b}$ = $\;10^{15\times 256}$, so the total multi-parameter key space for the MP-FrFT-OFDM is equal to $10^{7680}$. Finally, the total key space of the proposed cryptosystem can be calculated as $10^{60}\times 4\times 10^{5}{\times 10}^{7680}={4\times 10}^{7745}=2^{2200}$. The findings showed that the key space of our approach is very vast, preventing all sorts of brute force assaults. The key space of the proposed algorithm is more than $2^{100}$. The findings and analyses of the important space analysis are presented in Figure 10.

#### 5.2.5. Entropy Analysis

The unpredictability of the received image is calculated using entropy, which is a measure of uncertainty in the cyphered image. Strong randomness and strong confidentiality are signs that the encoded image has high entropy [13]. One definition of entropy in an information system reads like follows:

(32) $$H(m)=-\sum_{i=0}^{2^{N}-1}p(m_{i}){log}_{2}p(m_{i})$$

where “*m*” is the information source, the symbol “$m_{i}$” is represented by *N* total bits, it has a probability of $p_{\left(m_{i}\right)}$, and the optimal information entropy value is close to 8. The entropy result based on the proposed algorithm is 7.9999.

#### 5.2.6. Key Sensitivity Analysis

**Figure 11:**
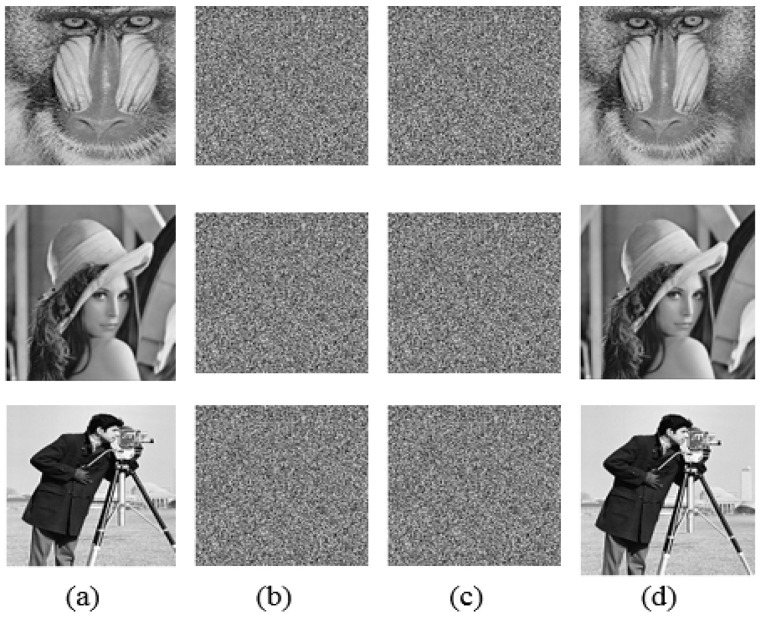
Key sensitivity analysis, original images are shown in (**a**), cipher images of the original key are shown in (**b**). Decrypted images for the incorrect decryption key are shown in (**c**), decrypted images for the correct decryption key are shown in (**d**).

A strong encryption system should be highly sensitive to even the smallest alteration to the secret keys [13]. Assume the control settings and beginning values that are used to encrypt plain photos $\left(x_{0}^{1}\right)$ and ($r^{1}$,$r^{2}$,…,$r^{4})$ in order to test the key sensitivity. Use the new key to decode the image after the encryption process by adding $10^{-16}$ to any beginning condition or control parameter. As a result, Figure 11 shows the key sensitivity test demonstrates how sensitive the proposed encryption system is to the security key. That indicates the least amount of secret key modification during the decoding procedure. The outcome will be an image that is entirely unencrypted.

## 6. Comparative Analysis

In this section, the performance comparison between the proposed cryptosystem results and other methods described in the literature for the Lena image of size 256 × 256 is shown in Table 14. The comparison between the proposed cryptosystem and the other recent methods is based on different criteria such as key space, entropy, correlation, NPCR and UACI. As shown in Table 14, whether the proposed DCBS-MP-FrFT-OFDM cryptosystem has the capacity to withstand various attacks is evaluated in order to determine the encryption system’s strength. The proposed method for evaluating it was put through a safety check, which included discussions of the histogram, entropy, correlation coefficient, NPCR, UACI, and NIST randomness tests.

## 7. Conclusions

A new physical layer authenticated encryption (PLAE) technique focused on the multi-parameter fractional Fourier transform–orthogonal frequency division multiplexing (MP-FrFT-OFDM) is proposed in this paper for secure image transmission over public IoT networks. This paper designs and studies a new, robust multi-cascaded chaotic modular fractional sine map (MCC-MF sine map). A novel dynamic chaotic biometric (Digital Fingerprint) signature (DCBS) generator based on combining the biometric signature and the suggested MCC-MF sine map random chaotic sequence output is also devised. It is based on the proposed MCC-MF sine map random chaotic sequence output. For the multi-parameter fractional Fourier transform in the OFDM system, which studies the encryption process in the frequency domain, the suggested DCBS generator’s output is used as a dynamic secret key. The suggested DCBS secret key generator is used to satisfy the secrecy and authentication features. The proposed DCBS-MP-FrFT-OFDM cryptosystem over IoT network’s security strengths are tested using statistical analysis, differential analysis, and key sensitivity analysis. The suggested proposed DCBS-MP-FrFT-OFDM cryptosystem’s ability to withstand various attacks is tested in order to gauge how strong the encryption system is. The suggested approach for evaluating it was subjected to a safety examination, which covered discussions of the histogram, entropy, correlation coefficient, NPCR, UACI, and NIST randomness tests.

This study adds to the body of literature by further examining the flaws of using the MPFrFT as two dimensions with multi-parameters of the FrFT, which increase the secret key space based on multi-phase shifting strategy in OFDM. The proposed DCBS-MP-FrFT-OFDM cryptosystem does not need any additional equipment, except the OFDM is replaced by MP-FrFT-OFDM and an external two-dimensional multi-parameters DCBS generator. The DCBS generator generates the all the secret keys in the proposed cryptosystem. In addition, the limitations of the proposed MPFrFT-OFDM scheme include that the two secret vectors can be optimized in order to improve the BER performance. On the other hand, for the security analysis, the MPFrFT OFDM has a very large key space as discussed in the key space analysis compared with other systems.

In the future, we will propose a brand-new deep CNN that can produce a digital signature in order to satisfy the identity property. Additionally, a digital deep CNN signcryption system can be created to combine the encryption and digital signature. Future studies could also concentrate on watermarking, data hiding in encrypted images, and stream video encryption and decoding. It will also be advised to use a new Deep Convolutional Neural Network for a compression–encryption system. Also, in future work, different optimization schemes can be used to optimize the selection of the two vectors $\bar{a}$ and $\bar{b}$ with a size of 1 × 256 fractional numbers in the range of (0 to 1) to improve the BER performance of the proposed MPFrFT OFDM.

## Figures and Tables

**Table 1:** The randomness tests results for the proposed MCC-MF sine map based on NIST SP800-22 tests.

Test	*p*-Value	Result
Monobit frequency	0.5961	Success
Block frequency	0.3673	Success
Runs test	0.7286	Success
Longest run of ones	0.8837	Success
Binary matrix rank test	0.2735	Success
Discrete Fourier transform	0.1942	Success
Non-overlapping template	0.3061	Success
Overlapping templates	0.7398	Success
Universal statistical	0.8394	Success
Linear complexity	0.7193	Success
Serial test	0.5037	Success
Approximate entropy	0.6695	Success
Cumulative sums (forward)	0.8359	Success
Cumulative sums (revere)	0.3891	Success
Random excursions	0.7291	Success

**Table 2:** The multi-secure parameters used in the simulations.

Parameter	Value
SK (Hex.)	4071A20C3CB340E95E65AF06549CCD16220A C0D998DEE50C22550EA6637F5AFA
$x_{0}$	0.918347421094373
$r_{1}^{f}$	10.485174284360704
$r_{2}^{f}$	18.936817384042791
$r_{3}^{f}$	9.195731663827418
$r_{4}^{f}$	3.038618376892133
$v_{1}$	0.843728376417384
$v_{2}$	0.172865272648265
$v_{3}$	0.447162948327648
$v_{4}$	0.728395273521837
a	a=256 bytes $\left(a_{1},a_{2},\cdots ,a_{256}\right)$
b	b=256 bytes $\left(b_{1},b_{2},\cdots ,b_{256}\right)$

**Table 3:** The proposed authenticated secure image transmission system simulation parameters.

Gray-Scale Image	Size	256×256
Channel coding	Type	Convolutional Encoder
	Code Rate	1/2
OFDM parameters	Sub-carrier ($N_{sc}$)	256
	FFT length	256
	Cyclic prefix (CP)	32
Attacks	AWGN	$\left(\begin{array}{c} \sigma^{2}=0.01,\;0.05,\\ 0.10,\;0.15,\;0.20 \end{array}\right)$
	Salt and Pepper noise	$\left(\sigma^{2}=0.02\right)$
	Speckle noise	$\left(\sigma^{2}=0.02\right)$
Key Performance Indicators (KPI)	Visual Quality Metrics (Clarity investigation)	$E_{b}N_{0}\;vs.BER$ $E_{b}N_{0}\;vs.PSNR$
	Encryption Quality Metrics (Statistical Analysis)	NPCR
		UACI
		$r_{xy}$, Histogram, Key space

**Table 4:** AWGN channel effect at zero mean μ=0 and over certain ranges of noise variances $\left(\sigma^{2}=0.01,0.05,0.10,0.15,0.20\right)$.

$\mathrm{Noise}\;\mathrm{Var}.\;(\sigma^{2})$		0.01	0.05	0.10	0.15	0.20
FFT	Decrypted Image	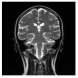	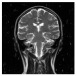	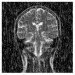	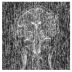	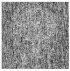
	PSNR(dB)	Inf	28.6397	14.515	8.8606	$6.56\times 10^{-2}$
FrFT	Decrypted Image	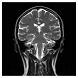	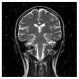	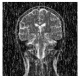	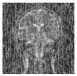	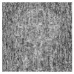
	PSNR(dB)	Inf	28.6397	14.515	8.8606	$6.56\times 10^{-2}$
MPFrTT	Decrypted Image	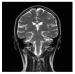	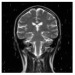	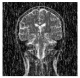	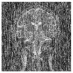	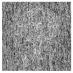
	PSNR(dB)	Inf	28.6397	14.515	8.8606	$6.56\times 10^{-2}$

**Table 5:** BER performances of the proposed FFT,FrFT and MPFrFT coded OFDM under Salt and Pepper noise, noise density d=0.02.

$E_{b}N_{0}$ (dB)	BER		
	FFT	FrFT	MPFrFT
0	0.1239	0.1117	0.1667
2	0.0314	0.0274	0.0484
4	0.0067	0.0053	0.0102
6	0.0017	0.0014	0.0022
8	$8.60\times 10^{-4}$	$8.21\times 10^{-4}$	$8.59\times 10^{-4}$
10	$7.59\times 10^{-4}$	$7.74\times 10^{-4}$	$7.58\times 10^{-4}$

**Table 6:** PSNR performances of the proposed FFT,FrFT and MPFrFT coded OFDM under Salt & Pepper noise, noise density d=0.02.

$E_{b}N_{0}\;(dB)$	PSNR (dB)		
	FFT	FrFT	MPFrFT
0	9.0709	9.5156	7.7798
2	15.0297	15.6164	13.1508
4	21.7511	22.6765	19.9282
6	27.5973	28.3270	26.6475
8	30.6554	30.8548	30.6579
10	31.1966	31.1123	31.2009

**Table 7:** BER and PSNR performance for FFT, FrFT, MPFrFT Coded OFDM over Salt and Pepper noise attack, d=0.02, at $E_{b}N_{0}=2,8,16dB$.

$E_{b}N_{0}\;(dB)$		2 dB	8 dB	16 dB
FFT	Decrypted Image	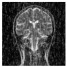	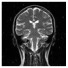	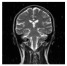
	BER	0.0314	$8.60\times 10^{-4}$	$7.58\times 10^{-4}$
	PSNR(dB)	15.0297	30.6554	31.2009
FrFT	Decrypted Image	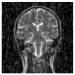	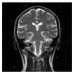	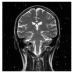
	BER	0.0274	$8.21\times 10^{-4}$	$7.58\times 10^{-4}$
	PSNR(dB)	9.5156	30.8548	31.2009
MP-FrFT	Decrypted Image	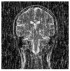	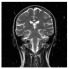	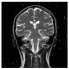
	BER	0.0484	$8.59\times 10^{-4}$	$7.58\times 10^{-4}$
	PSNR(dB)	13.1508	30.6579	31.2009

**Table 8:** BER and PSNR performances of the proposed FFT,FrFT and MPFrFT coded OFDM under Speckle noise attack; noise variance $\delta^{2}=0.02$.

$E_{b}N_{0}$ (dB)	BER			PSNR (dB)		
	FFT	FrFT	MPFrFT	FFT	FrFT	MPFrFT
0	0.1026	0.0920	0.1444	9.8868	10.3628	8.4049
2	0.0213	0.0178	0.0340	16.7095	17.5063	14.6790
4	0.0028	0.0020	0.0050	25.4816	27.0542	22.9900
6	$1.39\times 10^{-4}$	$8.58\times 10^{-5}$	0.0022	38.5823	40.6662	35.0743
8	$3.07\times 10^{-8}$	0	$3.11\times 10^{-4}$	75.1229	Inf	54.1514
10	0	0	$3.84\times 10^{-6}$	Inf	Inf	Inf

**Table 9:** BER and PSNR performance for FFT, FrFT, and MPFrFT Coded OFDM over Speckle noise attack, $\delta^{2}=0.02$, at $E_{b}N_{0}=2,8,8.50dB$.

$E_{b}N_{0}\;(dB)$		2 dB	8 dB	8.50 dB
FFT	Decrypted Image	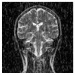	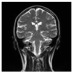	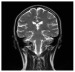
	BER	0.0213	$3.07\times 10^{-8}$	0
	PSNR(dB)	16.7095	75.1229	Inf
FrFT	Decrypted Image	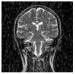	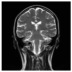	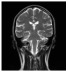
	BER	0.0178	0	0
	PSNR(dB)	17.5063	Inf	Inf
MP-FrFT	Decrypted Image	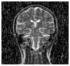	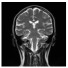	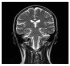
	BER	0.0340	$4.00\times 10^{-6}$	0
	PSNR(dB)	14.6790	54.1514	Inf

**Table 10:** NPCR comparison among the proposed MPFrFT and FrFT-coded OFDM using Cameraman, Peppers and Boat standard gray-scale test images.

Image	Encryption Scheme	
	MPFrFT	FrFT
Cameraman	99.6661	98.6773
Peppers	99.4720	98.3463
Boat	99.1598	99.0762

**Table 11:** UACI comparison among the proposed MPFrFT and FrFT-coded OFDM using Cameraman, Peppers and Boat standard gray-scale test images.

Image	Encryption Scheme	
	MPFrFT	FrFT
Cameraman	27.8645	26.8317
Peppers	24.0981	24.2064
Boat	21.8502	22.8703

**Table 12:** Correlation comparison among the proposed MPFrFT and FrFT-coded OFDM using Cameraman, Peppers and Boat standard gray-scale test images.

Image	Encryption Scheme	
	MPFrFT	FrFT
Lena	−0.0011	−0.0631
Cameraman	−0.0033	0.0012
Peppers	−0.00015	−0.0039
Boat	$4.11\times 10^{-4}$	0.0083

**Table 13:** Histogram analysis for the proposed MPFrFT-coded OFDM using Cameraman, Peppers and Boat standard gray-scale test images.

Test Image	Original Image Histogram	MPFrFT	
		Encryption	Decryption
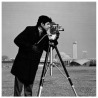	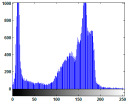	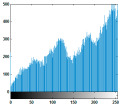	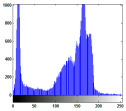
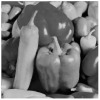	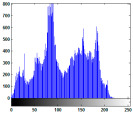	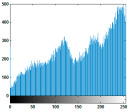	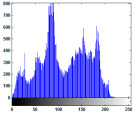
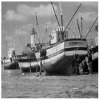	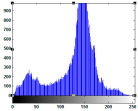	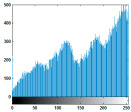	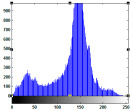

**Table 14:** Performance comparison between the proposed cryptosystem results and other methods described in the literature for a Lena image of size 256 × 256.

Criteria	Proposed	Ref. [48]	Ref. [74]	Ref. [75]	Ref. [76]	Ref. [77]
Key space	$2^{2200}$	-	$2^{942}$	$2^{441}$	-	-
Entropy	7.9999	7.7771	7.9997	7.9974	7.9022	7.9973
CC-H	−0.0011	-0.0219	−0.0002	0.0021	0.0022	0.0044
CC-V	0.0127	-	0.0004	-	-	
CC-V	0.0087	-	0.0001	-	-	
NPCR (%)	99.8945	99.7400	99.611	99.6123	99.62	99.63
UACI (%)	33.5283	27.5200	33.471	33.46	33.46	–
Authentication	✓	×	✓	×	×	×
Encryption	✓	✓	✓	✓	✓	✓

## Data Availability

Not applicable.
